# Intrapituitary mechanisms underlying the control of fertility: key players in seasonal breeding

**DOI:** 10.1016/j.domaniend.2016.01.002

**Published:** 2016-07

**Authors:** D.J. Tortonese

**Affiliations:** Centre for Comparative and Clinical Anatomy, Faculty of Health Sciences, University of Bristol, Bristol, UK

**Keywords:** Paracrinicity, GnRH, Gonadotropes, Lactotropes, Folliculostellate cells, Photoperiod

## Abstract

Recent studies have shown that, in conjunction with dynamic changes in the secretion of GnRH from the hypothalamus, paracrine interactions within the pituitary gland play an important role in the regulation of fertility during the annual reproductive cycle. Morphological studies have provided evidence for close associations between gonadotropes and lactotropes and gap junction coupling between these cells in a variety of species. The physiological significance of this cellular interaction was supported by subsequent studies revealing the expression of prolactin receptors in both the pars distalis and pars tuberalis regions of the pituitary. This cellular interaction is critical for adequate gonadotropin output because, in the presence of dopamine, prolactin can negatively regulate the LH response to GnRH. Receptor signaling studies showed that signal convergence at the level of protein kinase C and phospholipase C within the gonadotrope underlies the resulting inhibition of LH secretion. Although this is a conserved mechanism present in all species studied so far, in seasonal breeders such as the sheep and the horse, this mechanism is regulated by photoperiod, as it is only apparent during the long days of spring and summer. At this time of year, the nonbreeding season of the sheep coincides with the breeding season of the horse, indicating that this inhibitory system plays different roles in short- and long-day breeders. Although in the sheep, it contributes to the complete suppression of the reproductive axis, in the horse, it is likely to participate in the fine-tuning of gonadotropin output by preventing gonadotrope desensitization. The photoperiodic regulation of this inhibitory mechanism appears to rely on alterations in the folliculostellate cell population. Indeed, electron microscopic studies have recently shown increased folliculostellate cell area together with upregulation of their adherens junctions during the spring and summer. The association between gonadotropes and lactotropes could also underlie an interaction between the gonadotropic and prolactin axes in the opposite direction. In support of this alternative, a series of studies have demonstrated that GnRH stimulates prolactin secretion in sheep through a mechanism that does not involve the mediatory actions of LH or FSH and that this stimulatory effect of GnRH on the prolactin axis is seasonally regulated. Collectively, these findings highlight the importance of intercellular communications within the pituitary in the control of gonadotropin and prolactin secretion during the annual reproductive cycle in seasonal breeders.

## Highlights

• Paracrine interactions in the pituitary play a key role in the control of fertility.
• Gonadotroph-lactotroph crosstalk is bidirectional and seasonally regulated.
• Prolactin and dopamine inhibition of gonadotrophins is blocked by short days.
• Signal convergence at the level of protein kinase C and phospholipase C mediates prolactin-dopamine inhibition.
• Seasonal alterations in folliculostellate cells appear to modulate cellular crosstalk.

## 1 Introduction

It is well-accepted that the synthesis and release of gonadotropins is under hypothalamic regulation through the stimulatory and inhibitory actions of GnRH and gonadotropin-inhibitory hormone, respectively [1]. Notwithstanding, it has become apparent that, in addition to this hypothalamic regulatory system, the secretion of LH and FSH is also controlled by paracrine mechanisms that operate locally within the pituitary gland. In photoperiodic species, such as the sheep and the horse, temporal changes in GnRH and gonadotropin secretion during the annual reproductive cycle are controlled by seasonal changes in day length. Photoperiodic information is decoded by the pattern of nocturnal secretion of melatonin from the pineal gland; as melatonin synthesis is suppressed by light, its secretory pattern provides an index of night length. Critically, the pars tuberalis of the pituitary gland contains a high density of melatonin receptors, which on activation, regulate the annual pattern of prolactin secretion directly within the pituitary, that is, without the need of hypothalamic input [2]. This provides an intrapituitary regulatory system for hormone output that responds to an external independent cue (photoperiod) and implies that paracrine regulation of gonadotrope function by prolactin secreting cells can play a key role in the seasonal control of fertility.

## 2 Morphologic associations between the gonadotropic and lactotropic axes

**Fig. 1:**
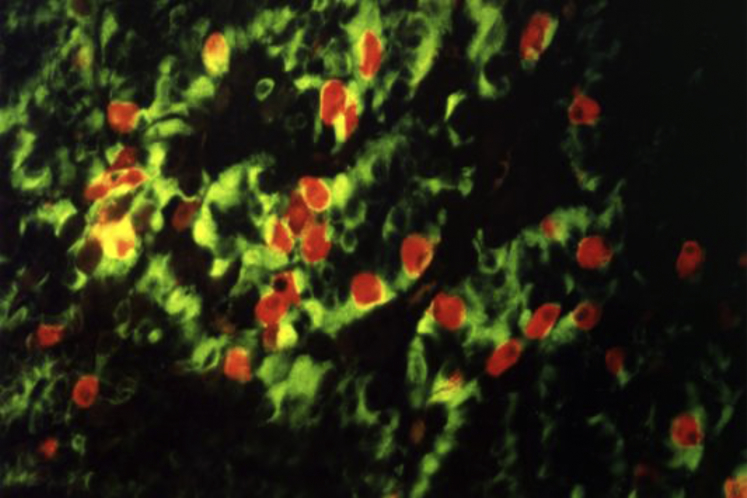
Double immunofluorescent staining for LHβ and prolactin in the pars distalis of the equine pituitary gland. A distinctive histologic arrangement between LH-gonadotropes (red) and lactotropes (green) where LH-secreting cells embedded within lactotrope clusters can be seen; magnification of ×200. (For interpretation of the references to color in this figure legend, the reader is referred to the Web version of this article.)

Adapted from Townsend et al, 2004 [7].

Microanatomical associations between gonadotrope and lactotrope cells were first described in the rat pituitary [3], [4] and then corroborated in larger vertebrates, including the rhesus monkey (D. Meeran, H. Urbanski, and D. Tortonese; unpublished) and seasonal breeders such as the sheep [5] and the horse [6], [7]. In this anatomic arrangement, gonadotropes are completely surrounded by cup-shaped lactotropes and intimately embedded within lactotrope clusters (Fig. 1). Although associations among other types of cells, for example between corticotropes and somatotropes, were also described in the pituitary gland of the rat, the gonadotrope/lactotrope interaction is most relevant for reproductive function and appears to be particularly important in photoperiodic species [8], [9], [10]. The incidence of gonadotropes is relatively constant throughout the year in the pars distalis [7], [11] and has been shown to be upregulated only in the pars tuberalis of sexually active females [10], [12]; conversely, the lactotrope population, which is only present in the pars distalis, undergoes dynamic changes in response to photoperiod. In sheep, the lactotrope cell area is larger in the nonbreeding season (summer) [13], [14], whereas in the horse, gonadal independent effects of season were reported in orchidectomized animals, with an increase in the incidence of lactotropes in the breeding season (summer) [7]. Moreover, the ultrastructure of lactotrope cells exhibits dynamic changes throughout the year in the ovine pituitary, with hypertrophy, increased cytoplasmic area, and increased rough endoplasmic reticulum and density of prolactin secretory granules during the long days of summer [13]. Similar ultrastructural changes in response to season were reported in another photoperiodic species, the white tail deer [15], [16]. Thus, although the gonadotrope population appears to be stable throughout the year in most species, seasonally regulated changes in the lactotrope population result in circannual alterations in the lactotrope/gonadotrope cell communication, which are modulated by gonadal feedback and are likely to affect the function of gonadotropes.

## 3 Physiological significance of gonadotrope/lactotrope associations

### 3.1 Lactotrope regulation of gonadotrope function

**Fig. 2:**
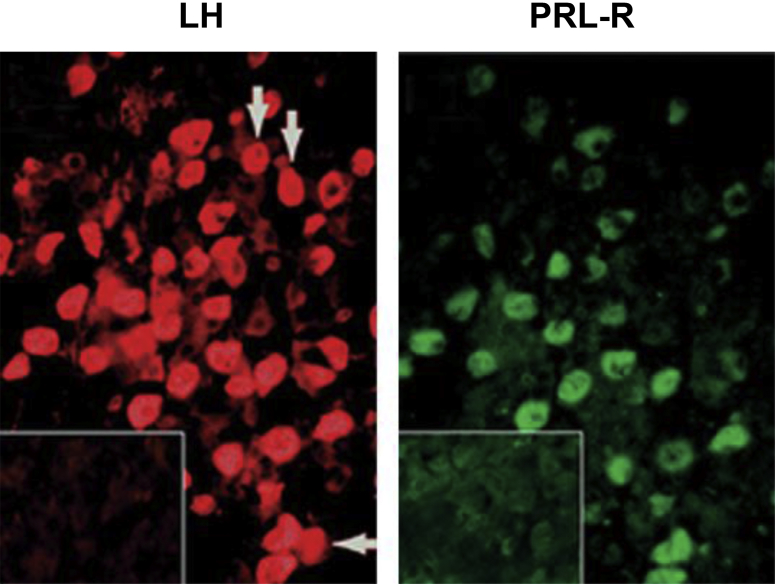
Double immunofluorescent staining for LHβ (left) and prolactin receptor (PRL-R; right) in the pars distalis of the ovine pituitary gland. Paraffin-embedded sections were incubated with a prolactin receptor polyclonal antibody which recognizes both the long and short forms of the receptor and a mouse monoclonal antibody specific to the LHβ-subunit. Note that the prolactin receptor is selectively expressed in the gonadotrope but that not all gonadotropes express the proalctin receptor (arrows). Inserts are the negative controls; ×200.

Adapted from Tortonese et al, 1998 [5].

**Fig. 3:**
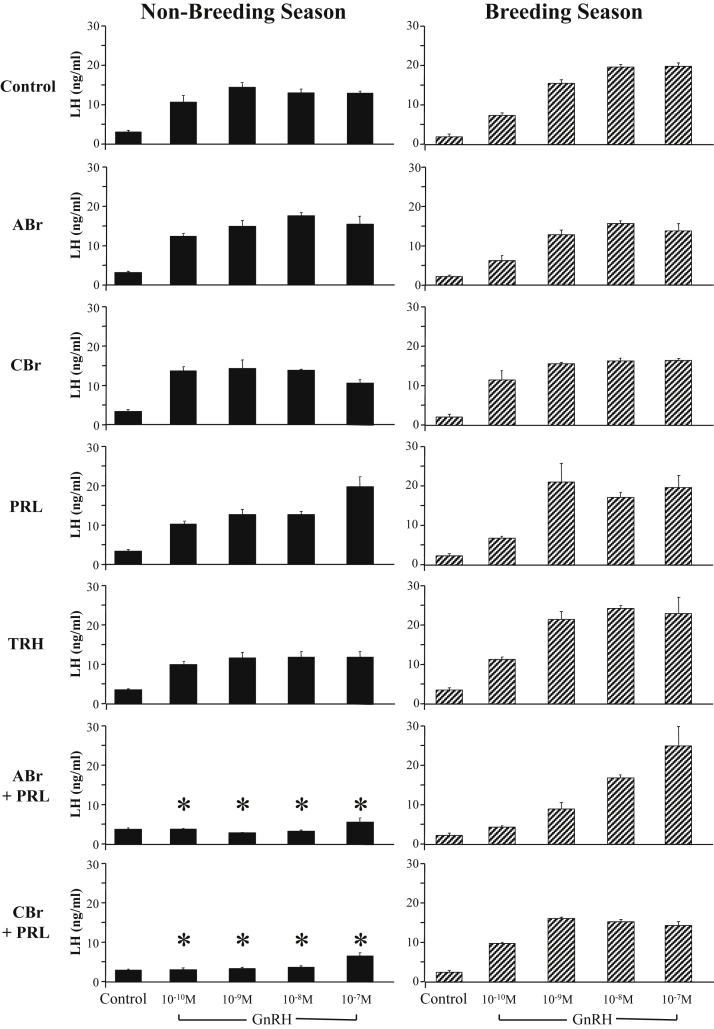
LH response to GnRH in ovine pituitary primary cell cultures during the nonbreeding season (NBS; left) and breeding season (BS; right) after treatments designed to increase or decrease the concentrations of prolactin in the culture. Treatments were as follows: (1) medium (Control), (2) acute (90-min) bromocriptine (ABr), (3) chronic (7-d) bromocriptine (CBr), (4) ABr plus prolactin (ABr + PRL), (5) CBr plus PRL (CBr + PRL), (6) PRL, or (7) thyrotropin-releasing hormone (TRH). The LH response to increasing concentrations of GnRH (from 0 to 10^–^^7^ M) is shown for each experimental treatment group. Each bar represents the mean ± standard error of the mean. Note the following: (1) A classical dose response to increasing concentrations of GnRH was observed in the control groups, where only medium and GnRH were applied; (2) administration of prolactin and a dopamine agonist (ABr + PRL and CBr + PRL) resulted in a highly significant suppression of LH release at all concentrations of GnRH; (3) this effect was seasonally regulated, as it was only apparent during the nonbreeding season (summer); and (4) no significant difference in the GnRH-stimulated LH release was observed in response to any of the other treatments. *P < 0.01 vs same dose of GnRH in the Control group.

Adapted from Gregory et al, 2004 [22].

**Fig. 4:**
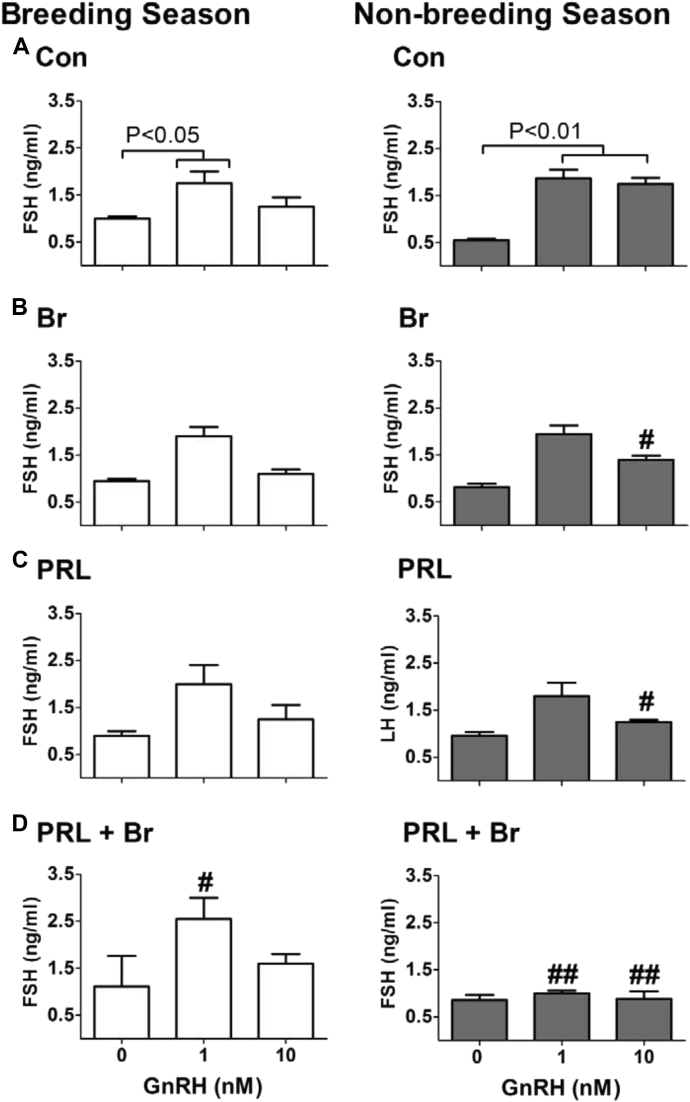
FSH response to GnRH in ovine pituitary primary cell cultures during the breeding season and nonbreeding season after treatments designed to increase or decrease the concentrations of prolactin in the culture. Treatments were as follows: (A) medium (control; Con), (B) bromocriptine (Br), (C) prolactin (PRL), and (D) prolactin plus Br plus (PRL + Br). The FSH response to GnRH administered at concentrations of 0, 1, and 10 nM is shown for each experimental treatment group during the breeding season and the nonbreeding season. Each bar represents the mean ± standard error of the mean. Note that prolactin and the dopamine agonist (Br) suppressed the FSH-secretory response to GnRH in a photoperiod-dependent manner, as this effect was only apparent during the nonbreeding season (summer). ^#^*P* < 0.05 and ^##^*P* < 0.01 vs same dose of GnRH in Con group.

Adapted from Hodson et al, 2012 [23].

The most plausible mediator of the effects of lactotrope cells on gonadotrope function is their primary secretory product, that is, prolactin. Such an effect would require the presence and activation of prolactin receptors within the pituitary gland. Indeed, prolactin receptor messenger RNA (mRNA) expression was first detected in the rat [17] and then identified in the sheep [5] and horse [18] pituitaries. Importantly, translation of the signal into the long and short forms of the prolactin receptor protein was demonstrated in both these seasonal breeders [5], [6]. Moreover, in the sheep, but not in the horse, the expression was shown to be selectively confined to the gonadotrope (Fig. 2). Prolactin receptor expression was also reported in the mouse-derived gonadotrope cell lines αT3 and LβT2 [19]. The functional significance of the expression of prolactin receptors in the pituitary gland was investigated using ovine primary pituitary cell cultures obtained in the breeding season and nonbreeding season. Treatments designed to reduce or increase the concentrations of prolactin in the culture were unable to affect the LH response to GnRH; but the combined application of prolactin and a dopamine agonist (bromocriptine) completely blocked the LH response to the secretagogue. This inhibitory system proved to be conserved across species because it was also present in the horse and detectable in mouse-derived gonadotrope cell lines [19], [20], [21]. Critically, the combined suppressive effect of prolactin and dopamine was shown to be seasonally regulated, as it was only apparent in the summer, during the ovine nonbreeding season [22], [23] (Fig. 3). Moreover, in the horse it was also shown to be seasonally regulated, with suppression of the LH response to GnRH occurring only in the summer, that is, the equine breeding season [20]. It is important to note that the secretion of prolactin in photoperiodic species is upregulated under the long days of summer, irrespective of whether the animals are long- or short-day breeders [24], [25], [26] and that the activity of hypothalamic dopaminergic networks is also increased under long days [27]. As the combined inhibitory actions of prolactin and dopamine on GnRH-stimulated gonadotropin secretion occurred at opposite stages of the ovine and equine annual reproductive cycles, these factors must play different roles in short- and long-day breeders. In the former, these contribute to the complete suppression of the reproductive axis during the nonbreeding season, whereas in the latter, the same are likely to fine-tune the gonadotrope responsiveness to GnRH to prevent desensitization of the GnRH receptor [28] and to contribute to the differential regulation of LH and FSH secretion. Indeed, the combined inhibitory effects of prolactin and dopamine were also apparent on the FSH response to GnRH in sheep [23] (Fig. 4), corroborating complete inhibition of the gonadotropic axis, whereas no effects on FSH were observed in the horse although the LH response was suppressed [20].

Hyperprolactinemia, whether experimentally induced [29], [30], lactational [31], [32], [33] or pathological [34], is known to suppress gonadotropin secretion in rodents and primates. In humans, hypersecretion of prolactin resulting from a pituitary prolactinoma is a major cause of amenorrhea in women and impairs fertility in men [35], [36], but the specific mechanisms underlying these inhibitory effects on fertility remain unresolved. In sheep, administration of thyrotropin-releasing hormone (TRH), a potent stimulator of prolactin secretion, disrupted the estradiol-induced preovulatory surge of LH [37]. This effect could be due to the stimulation of prolactin by TRH and suppression of GnRH at the level of the hypothalamus, as functional prolactin receptors have been reported in a subpopulation of GnRH neurons [38], and prolactin was shown both to reduce the content of GnRH in portal blood [39] and to affect hypothalamic networks known to regulate GnRH neurons [40], [41], [42]. However, the LH response to GnRH was impaired by prolactin in rodents [43], [44], indicating that prolactin also acts at the level of the pituitary to suppress gonadotropin secretion. Critically, in seasonal breeders, prolactin inhibition of gonadotropin secretion at the level of the pituitary only occurs in conjunction with dopamine. This potent inhibitory mechanism regulates not only hormone release but also gonadotropin synthesis, as the *LH* mRNA response to GnRH was also blocked by the combined actions of prolactin and dopamine [23]. However, the seasonal regulation of this inhibition appears to be exerted at the level of hormone release because *LH* gene expression was suppressed in both the breeding and nonbreeding season [23].

Melatonin relays the effects of photoperiod on the prolactin axis through an action exerted at the level of the pituitary gland via the activation of melatonin receptors in the pars tuberalis [2]. This region does not contain lactotropes in the ovine pituitary [5], [45], implying that a paracrine mechanism is likely to mediate the melatonin-induced suppression of prolactin. Interestingly, our studies have revealed that the pars tuberalis is needed for the photoperiodic regulation of the suppressive actions prolactin and dopamine on the FSH response to GnRH, but not for the response of LH. This indicates an essential communication between the pars tuberalis and the pars distalis for the differential control of gonadotropin secretion, which is known to be vital for fertility [46], [47]. It should be noted that the suppression of gonadotropin output by prolactin and dopamine also occurs in mouse-derived gonadotropes and that in seasonal breeders this inhibition only takes place during the long days of summer when the nocturnal melatonin peak is of short duration. Therefore, the blockade of this inhibition by the long duration of the nocturnal melatonin peak in the short days of winter constitutes an active regulatory system, whereas the combined effects of prolactin and dopamine to downregulate gonadotropin output can be considered the default mechanism. Our studies have shown that, in photoperiodic species, the blockade of this mechanism operates locally within the pituitary in response to an external independent cue (photoperiod) to control seasonal reproduction.

### 3.2 Intracellular signaling pathways mediating prolactin and dopamine inhibition of the gonadotrope response to GnRH

**Fig. 5:**
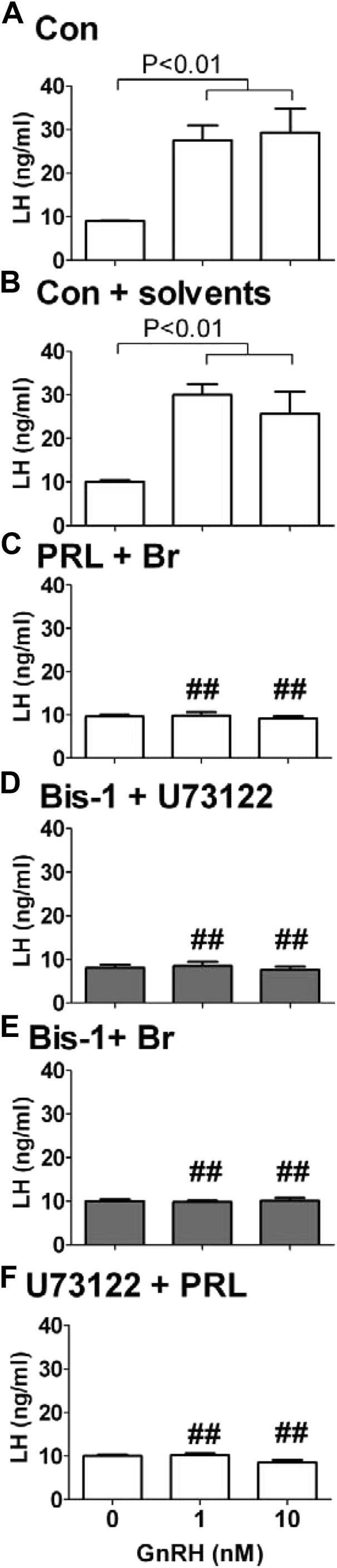
Signaling convergence at the levels of protein kinase C (PKC) and phopholipase C (PLC) is required for the suppression of the LH response to GnRH by prolactin and dopamine. Luteinizing hormone release from ovine pituitary primary cultures during the nonbreeding season after treatment with: (A) medium (Con); (B) medium + solvents used to dilute PKC and PLC inhibitors (Con + solvents); (C) prolactin + bromocriptine (PRL + Br); (D) Bis-1 + U73122 (specific inhibitors of PKC and PLC, respectively); (E) Bis-1 + Br; and (F) U73122 + PRL. The LH response to GnRH administered at concentrations of 0, 1, and 10 nM is shown for each experimental treatment group. Each bar represents the mean ± standard error of the mean. Note that the suppression of the LH response to GnRH by prolactin and the dopamine agonist bromocriptine is mimicked by the application of the PKC and PLC inhibitors and undistinguishable from that resulting from the combined application of the PKC inhibitor and the dopamine agonist or the PLC inhibitor and prolactin. ^##^*P* < 0.01 vs same dose of GnRH in Con group.

Adapted from Hodson et al, 2012 [23].

The intracellular mechanism underlying the combined inhibitory actions of prolactin and dopamine on gonadotropin output could rely on crosstalk among the signaling pathways activated by the binding of GnRH, prolactin, and dopamine to their respective cognate receptors. Binding of GnRH to its G-protein-coupled receptor activates Gq and/or G11 proteins and stimulates phospholipase C (PLC), leading to the production of diacylglycerol and activation of protein kinase C (PKC) isoforms before inducing calcium mobilization [48], [49]. Gonadotropin-releasing hormone also activates the extracellularly regulated kinase cascade of the mitogen-activated protein kinase (MAPK)–signaling pathway [50]. Prolactin binding to its cytokine-type receptor leads to phosphorylation of the tyrosine kinase JACK2 and subsequent phosphorylation and activation of STAT5, but the stimulation of MAPK cascades and interaction between these and the JAK2-STAT5 pathway are also known to occur [51], [52]. As the dopamine D2 receptor is coupled to inhibitory G proteins (Ga0 and/or Ga1), activation of this receptor will result in inhibition of adenylyl cyclase, cyclic adenosine monophosphate, protein kinase A, PLC, and PKC [53], [54]. Therefore, PLC and PKC are common pathways in the signaling cascade–mediating activation of the GnRH, prolactin, and dopamine receptors. In seasonal breeders, we have shown that neither prolactin nor dopamine suppressed gonadotropin secretion when given separately. Using specific antagonists to PKC and PLC, we found that, in agreement with those results, the ovine LH response to GnRH was not affected by the single application of either compound. However, the secretion of LH in response to the decapeptide was blocked when the antagonists were applied simultaneously, and these effects were undistinguishable from those resulting from the combined application of prolactin and dopamine [23] (Fig. 5). This indicates that stimulation of LH secretion by GnRH is only inhibited when PKC and PLC signaling pathways are concomitantly downregulated by prolactin and dopamine. The signaling crosstalk between PKC and PLC cascades underlying the inhibitory effects of prolactin and dopamine on gonadotropin secretion provides a target for the photoperiodic blockade of this mechanism by the long duration of nocturnal melatonin output during the short days of winter.

### 3.3 Gonadotrope regulation of lactotrope function

The associations between gonadotropes and lactotropes in the pars distalis of the pituitary also provide the morphological basis for an interaction between the gonadotropic and prolactin axes in the reversed direction, that is, the regulation of prolactin cells by gonadotropin-secreting cell populations. Indeed, GnRH has been shown to stimulate prolactin secretion in a variety of species including rodents and humans [55], [56], [57]. In these studies, however, specific paracrine regulation within the pituitary could not be determined, but the comprehensive work by Carl Denef and coworkers clearly demonstrated that gonadotrope cells mediate the stimulatory effects of GnRH on prolactin secretion [9]. A series of studies showed that the sustained increase in prolactin output observed in rat pituitary cultures after treatment with GnRH was obliterated in lactotrope-enriched cell populations [58]. Conversely, the addition of αT3–1 gonadotrope cells, which are known to express the GnRH receptor, to lactotrope-enriched cultures restored the ability of GnRH to stimulate prolactin secretion [59]. Moreover, conditioned media recovered from GnRH–stimulated gonadotrope-enriched aggregates readily stimulated prolactin release from lactotrope-enriched cultures [58]. These studies clearly show that not only can the gonadotrope cell stimulate lactotrope function and mediate the effect of GnRH on prolactin secretion, but also that this action results from a paracrine mechanism involving a gonadotrope secretory product.

**Fig. 6:**
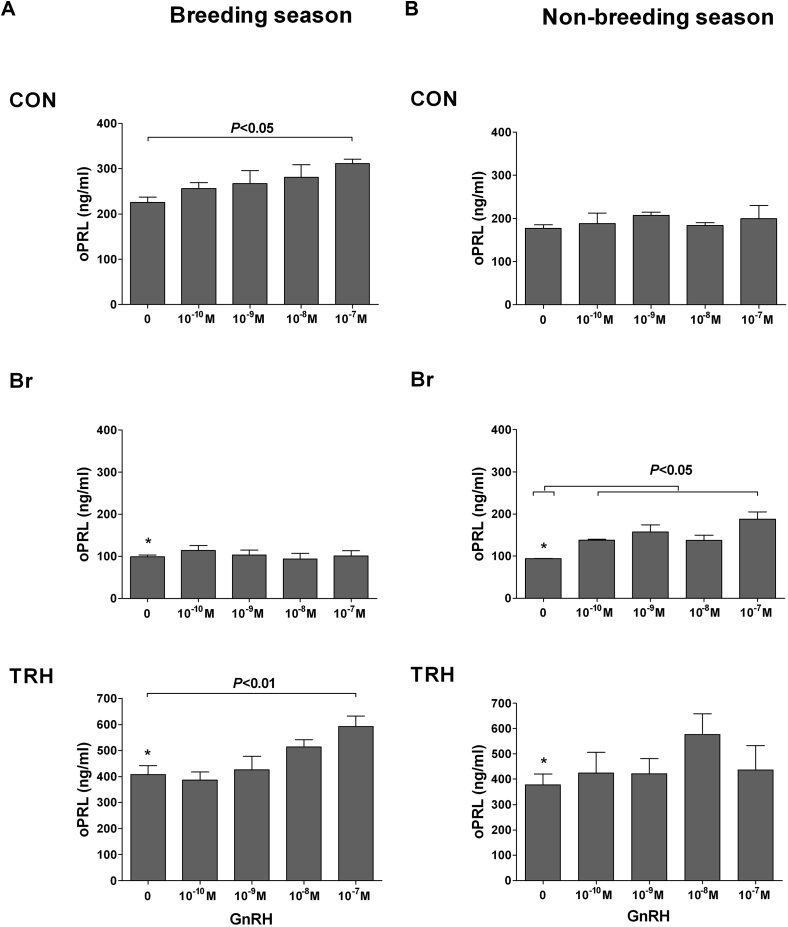
Seasonal modulation of the stimulatory effects of GnRH on prolactin secretion. Prolactin response to increasing concentrations (0 to 10^–^^7^ M) of GnRH in ovine pituitary primary cell cultures during the breeding season (A) and nonbreeding season (B) after the treatment with: (1) medium alone (CON), (2) bromocriptine (Br), and (3) thyrotropin-releasing hormone (TRH). Each bar represents the mean + standard error of the mean. The Y-axes have been adjusted for the TRH experimental group to account for the magnitude of the response. Note the following: (1) GnRH stimulated prolactin release in the breeding season but not in the nonbreeding season; (2) the dopamine agonist Br suppressed basal prolactin concentrations in both the breeding season and nonbreeding season and inhibited the prolactin response to GnRH in the breeding season; (3) treatment with Br in the nonbreeding season resulted in GnRH-stimulating prolactin secretion; and (4) TRH stimulated basal prolactin secretion in both the breeding season and nonbreeding season and enhanced the prolactin response to GnRH in the breeding season. *P* < 0.05 and *P* < 0.01 for differences with 0-M GnRH within treatment group; *P < 0.01 for differences with CON within season.

Adapted from Henderson et al, 2008 [18].

In seasonal breeders, we have shown that gonadotrope effects on lactotrope function are also apparent. Indeed, GnRH unequivocally stimulated prolactin release in ovine pituitary cultures in a dose-dependent manner [18]. This action of GnRH was blocked by the dopamine agonist bromocriptine and enhanced by the application of TRH. Importantly, the prolactin response to GnRH was shown to be seasonally regulated because it was observed only in cultures produced in the winter during the breeding season. Moreover, bromocriptine restored the ability of GnRH to stimulate prolactin release in cultures generated in the summer during the nonbreeding season, whereas the enhancement of the prolactin response to GnRH induced by TRH was only detected during the breeding season (Fig. 6). Additional studies demonstrated that the stimulatory actions of GnRH on prolactin output could not be mediated by the primary secretory products of the gonadotrope, that is, the gonadotropins, because neither the LH receptor nor the FSH receptor was expressed in the ovine or equine pituitary [18]. As GnRH receptors in the sheep pituitary gland are only expressed in gonadotrope cells, the GnRH effects on prolactin secretion must be mediated by another gonadotrope secretory product. In rodents, the α-gonadotropin subunit, epidermal growth factor, transforming growth factor-α, and proopiomelanocortin-derived products have all been proposed as plausible candidates to mediate the effects of GnRH on lactotrope function because these factors are produced by gonadotrope cells and stimulate prolactin production [9]. The physiological significance of the gonadotrope stimulation of lactotrope function during the short days of winter, in the ovine breeding season, remains to be elucidated. As at this time of year, prolactin alone was unable to affect the gonadotropin response to GnRH and the activity of hypothalamic dopaminergic neurons is reduced [22], [27], it is unlikely that the GnRH stimulation of prolactin will have direct inhibitory effects on gonadotropin output. However, it is possible that simultaneous stimulation of LH and prolactin by GnRH at this stage of the annual reproductive cycle will have a modulatory role in the feedback effects of gonadal steroids on gonadotrope function.

## 4 Paradoxical stimulatory effects of prolactin on gonadotrope cells

**Fig. 7:**
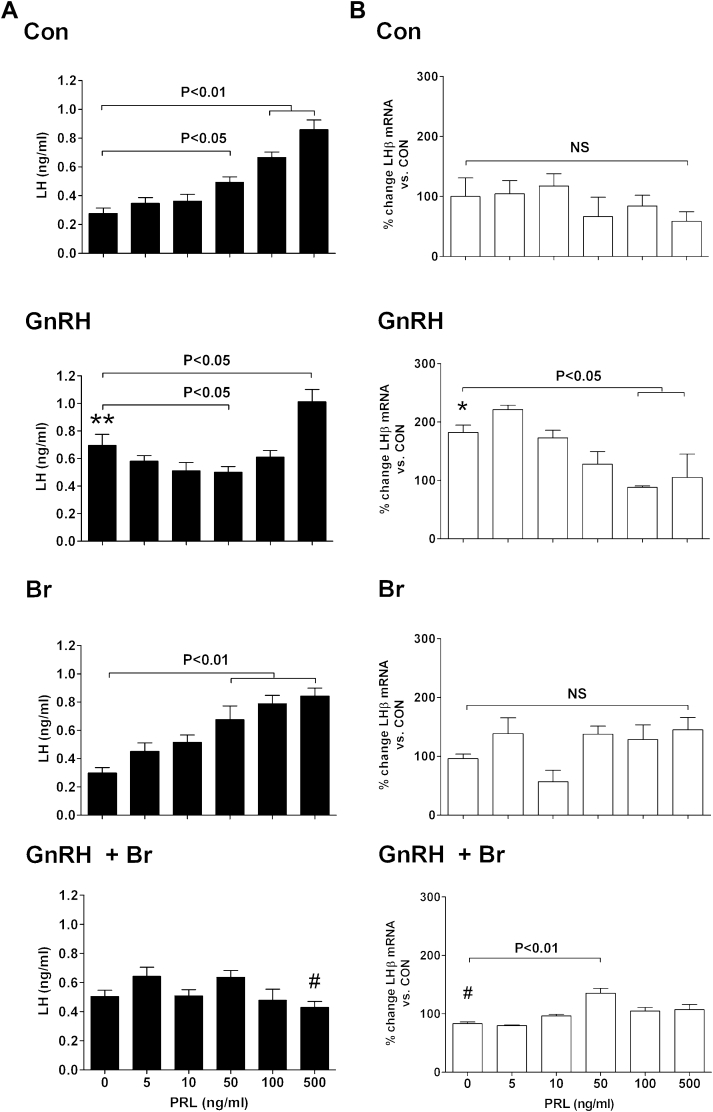
Effects of prolactin on basal- and GnRH-stimulated LH synthesis and release in gonadotrope cells. LβT2 gonadotropes were treated with increasing doses of prolactin in the presence of: (1) medium-alone (control, CON); (2) 10^−7^-M GnRH; (3) 10^−8^-M bromocriptine (Br); or (4) 10^−7^-M GnRH +10^−8^-M Br. The secretory (A) and messenger RNA (mRNA) (B) responses to treatments were measured. Note that, (1) Prolactin significantly increased basal LH secretion at concentrations of 50 ng/mL and higher under control (CON) conditions; (2) Prolactin blocked the LH response to GnRH at a dose of 50 ng/mL and enhanced it at a dose of 500 ng/mL; (3) The dopamine agonist Br reduced the LH response to GnRH alone and abolished the prolactin-induced biphasic modulation of the LH response to GnRH; (4) Prolactin had no effect on basal LHβ mRNA expression; (5) the LHβ mRNA response to GnRH was abolished by prolactin at concentrations of 100 ng/mL and higher; and (6) Br suppressed the LHβ mRNA response to GnRH in the absence of proalctin and allowed GnRH to stimulate LHβ gene expression in the presence of proalctin. Values represent the mean ± standard error of the mean. (*P < 0.05 and **P < 0.01 vs CON; ^#^*P* < 0.01 vs GnRH).

Adapted from Hodson et al, 2010 [21].

As previously mentioned, the inhibitory actions of prolactin on the gonadotropic axis at the level of the pituitary in rodents are well documented. Experimentally induced hyperprolactinemia led to a significant reduction in the proportion of LH-secreting cells [60] and impaired the postcastration increase in pituitary GnRH receptors [61], whereas in vitro treatments with prolactin suppressed both basal and GnRH-stimulated LH secretion from pituitary fragments [43]. However, in gonadotrope monocultures, we have shown that prolactin has a paradoxical stimulatory effect on LH release. Indeed, prolactin stimulated LH output in a dose-dependent manner in LβT2 gonadotrope cells and the effect was not impaired by the simultaneous application of a dopamine agonist [21] (Fig. 7). Moreover, although blockade of GnRH receptors by the GnRH agonist buserelin prevented the stimulation of LH release by the decapeptide, it enhanced the actions of prolactin on LH output. This stimulatory effect was exerted only on hormone release and not on hormone synthesis, as *LH* gene expression was not affected by prolactin at any of the doses tested. Subsequent intracellular signaling studies showed that the stimulatory actions of prolactin on LH release are mediated by a JAK2-PIK3-PKC–dependent signaling cascade, rather than by regular cytokine receptor pathways [21].

**Fig. 8:**
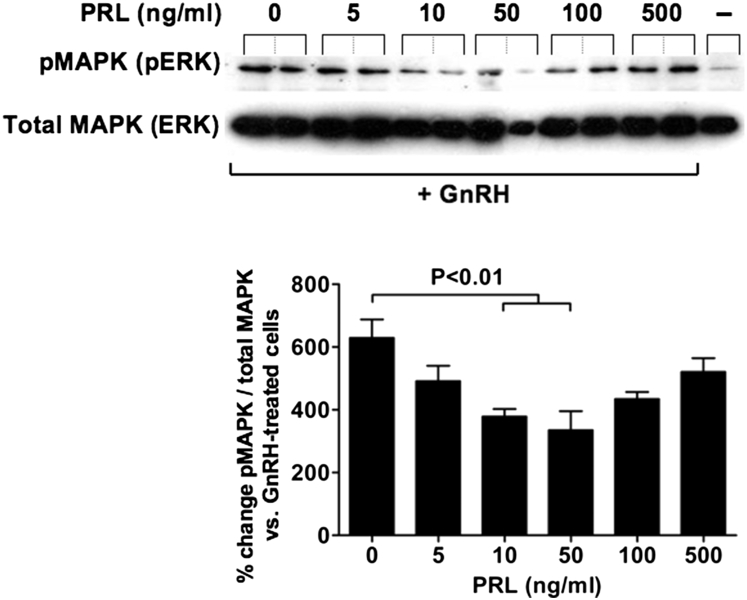
Effects of prolactin on GnRH-induced MAPK (ERK) phosphorylation in gonadotope cells. LβT2 gonadotropes were treated with increasing does of prolactin in the presence of 10^−7^-M GnRH and phosphorylated MAPK3/1 (pMAPK), and total MAPK (ERK) were assessed using Western blotting. Note that GnRH was able to invert the biphasic effect of prolactin receptor (PRL) on MAPK (ERK) phosphorylation. Note the biphasic effect of increasing doses of prolactin on GnRH-induced MAPK (ERK) phosphorylation. Values represent the mean ± standard error of the mean for at least 3 independent experiments. GnRH-treated (+) and untreated (−) controls were loaded to validate the antibody specificity. ERK, extracellularly regulated kinase; MAPK, mitogen-activated protein kinase; pERK, phosphorylated extracellular signal-regulated kinase; pMAPK, phosphorylated MAPK3/1.

Adapted from Hodson et al, 2010 [21].

These paradoxical effects of prolactin on the LH axis were recorded in the absence of GnRH. However, when gonadotropes were stimulated with a physiological dose of the decapeptide, the LH response was modulated by prolactin in a dose-dependent manner, resulting in a biphasic profile [21] (Fig. 7). The suppression of LH release was only observed at physiological doses of prolactin. This inhibitory effect was shown to result from the ability of prolactin to impair GnRH-induced MAPK phosphorylation (Fig. 8). Importantly, the biphasic modulation of the LH response to GnRH by increasing doses of prolactin proved to operate exclusively at the level of hormone release because the *LHβ* mRNA response to the secretagogue was blocked by prolactin at all doses tested. Thus, it appears that paradoxical stimulatory effects of prolactin on LH occur only at the level of hormone release and are only apparent when gonadotropes are deprived from contacts with other cell types and not stimulated by GnRH. Although such a situation is unlikely to occur in vivo, the results highlight the importance of heterologous intercellular contacts and secreatagogue stimulation for normal function of pituitary cells involved in reproduction.

## 5 Intercellular contacts underlying paracrine regulation of pituitary function during the annual reproductive cycle: role of the folliculostellate cell

**Fig. 9:**
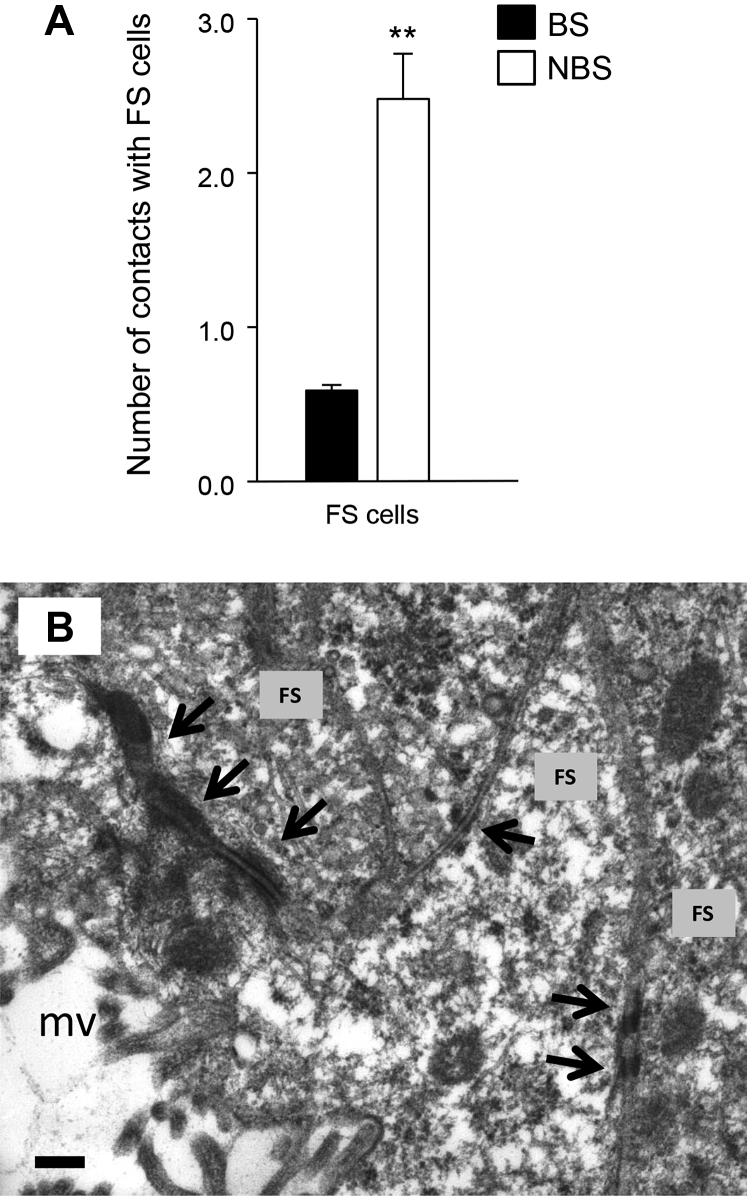
Folliculostellate (FS) cells junctional contacts in the pars distalis of the ovine pituitary gland. (A) Seasonal effects on the number of junctional contacts between FS cells in the pars distalis of the ovine pituitary gland. (B) Electron micrograph depicting the ultrastructure of adherens junctions between FS cells. Data are mean ± standard error of the mean; **P < 0.01. Scale bar = 200 nm. Arrows indicate adherens junctions. BS, breeding season (filled column); NBS, nonbreeding season (open column); mv, microvilli.

Adapted from Christian et al, 2015 [13].

The striking difference in the gonadotrope response to prolactin between in vivo, ex vivo, or in vitro studies in rodents in which multiple pituitary cell types were present and studies using gonadotrope monocultures points to a role of heterologous intercellular contacts in the physiological regulation of the pituitary mechanisms underlying the control of fertility. In long- and short-day seasonal breeders, we have shown that although the gonadotrope cell population in the pars distalis remains relatively constant throughout the annual reproductive cycle, with little or no change in gonadotrope subtypes, incidence, and intergonadotrope contacts [7], [12], [13], [62], the lactotrope cell population displays increased intercellular contacts and gonadal-independent enhanced cellular prevalence during the long days of summer [7], [13]. The upregulation in lactotrope intercellular communication was shown to be accompanied by changes in cellular ultrastructure, with increased rough endoplasmic reticulum, secretory granule density, and total cell area at this time of year. Although the gonadotrope and/or lactotrope intercellular contacts did not appear to change across the annual reproductive cycle, the increased communication among lactotropes is likely to play a role in the paracrine regulation of gonadotrope function. It is highly plausible that this intercellular interplay is modulated by folliculostellate cells. Folliculostellate cells are nonendocrine, star-shaped, glial-like cells, which organize themselves into follicles and communicate with each other and with endocrine cells through gap-junctions, generating a 3-dimensional network for the transmission of signals throughout the pituitary to coordinate its function [9], [63], [64], [65]. These cells produce and secrete a number of paracrine factors, which are known to influence both gonadotropes and lactotropes. Thus, although the folliculostellate cell production of follistatin inhibits FSH secretion through its ability to neutralize activin and thus plays a key role in the differential control of gonadotropin secretion, the production of interleukin-6, nitric oxide, and vascular endothelial growth factor (VEGF) regulates prolactin secretion [9], [66]. In seasonal breeders, folliculostellate cells have been shown to be conspicuously distributed throughout the pars distalis and pars tuberalis [18] and to respond to changes in photoperiod with remarkable plasticity in both long- and short-day breeders [67], [68]. As these cells do not appear to contain melatonin receptors, the reported effects of photoperiod and exogenous melatonin on this cell network are likely to be mediated by melatonin-responsive pars tuberalis specific cells. In a recent study, we have shown that folliculostellate cells in the ovine pituitary display overt ultrastructural changes throughout the annual reproductive cycle with increased cell size, greater amount of rough endoplasmic reticulum, and enhanced number of intercellular adherens junctions during the long days of summer, in the nonbreeding season [13]; (Fig. 9). At this time of year, these cells also showed dramatic changes in morphology, with an increased number of elongated processes surrounding endocrine cell clusters and upregulation of microvilli-lined follicles. Subsequent studies revealed that folliculostellate cells respond to the photoperiod and/or melatonin signal of the nonbreeding season in this species (sheep) by altering their production of specific VEGF isoforms and that this humoral response controls lactotrope and gonadotrope function [66] and appears to have autoregulatory effects on the folliculstellate cells of the pars distalis via activation of VEGF receptor-2 (J. Castle-Miller, D. Bates, and D. Tortonese; unpublished). As the overall upregulation of folliculostellate cell networks was found in both short-day breeders (sheep and mink) and long-day breeders (viscacha) at the same time of year (spring/summer), being at opposite stages of their annual reproductive cycles, it becomes apparent that these cells respond to the photoperiodic signal in similar ways in both short-day and long-day breeders but to generate different outcomes. Thus, it is possible that although in short-day breeders they participate in the transmission of photoperiodic information to regulate prolactin secretion and allow complete suppression of the gonadotropic axis at a time when the endogenous dopaminergic tone is high, in long-day breeders their primary function may be associated with the differential control of LH and FSH secretion and the fine-tuning of the gonadotrope responsiveness to GnRH.

## 6 Conclusions

**Fig. 10:**
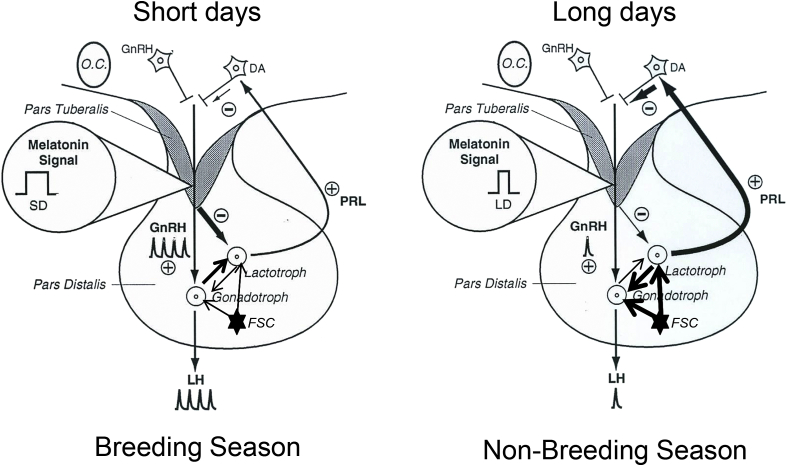
Intrapituitary control of seasonal breeding: a working model. Working hypothesis for the photoperiodic regulation of the intrapituitary control of gonadotropin and prolactin secretion during the annual reproductive cycle in sheep. The proposed interactions between gonadotrope and lactotrope cells and the modulatory role of folliculostellate cells in response to the pineal melatonin signal are depicted under both short days and long days. Under short days, the long duration of the nocturnal melatonin peak acting on melatonin-sensitive cells in the pars tuberalis (shaded area) suppresses prolactin secretion from the pars distalis through a paracrine mechanism, leading to reduced inhibition of gonadotrope function and downregulation of the modulatory actions of folliculostellate cells. The reduction in prolactin output also leads to reduced activation of inhibitory dopaminergic (DA) networks in the hypothalamus, which are known to suppress not only prolactin secretion but also the activity of GnRH neurons. The ensuring derepression of GnRH neurons results in increased frequency of GnRH release with the subsequent activation of gonadotropin secretion characteristic of the breeding season. This increase in GnRH output also acts on lactotrope cells via a paracrine mechanism mediated by the gonadotrope, possibly to enhance the crosstalk between these 2 cell types and modulate the gonadotrope response to gonadal feedback signals. Under long days, the short duration of the nocturnal melatonin peak fails to suppress lactotrope function leading to a dramatic increase in prolactin secretion, activation of dopamine neurons, downregulation of GnRH secretion, and complete suppression of the gonadotrope axis by the combined actions of prolactin and dopamine under enhanced activity of folliculostellate cell networks. This results in the suppression of fertility characteristic of the nonbreeding season. The size of the arrows denotes the activity of the system. FSC, folliculostellate cells; LD, long days; OC, optic chiasm; PRL, prolactin receptor; SD, short days.

It has become clearly apparent that the pituitary gland is not a slave organ controlled exclusively by hypothalamic inputs and peripheral tissue feedback, as it was originally believed, but that it can regulate its own function, and in turn those of body systems, through strategic communication of intrinsic cell networks. For the reproductive axis, intercellular communication between gonadotropes and lactotropes, and the modulation of this cellular crosstalk by folliculostellate cells, plays a key role in the mechanisms underlying temporal changes in fertility. The intimate cellular associations between gonadotropes and lactotropes and the presence of prolactin receptors in gonadotropin secreting cells provide the morphological basis for the paracrine regulation of the gonadotrope response to GnRH by prolactin. This regulatory system requires the participation of dopamine and is mediated by a PKC-PLC–signaling cascade. Paracrine regulation of these two cell types is also apparent in the opposite direction because GnRH can stimulate prolactin production through a gonadotrope-mediated mechanism. In photoperiodic species, the intercellular communication between gonadotropes and lactotropes is modulated by the suppressive actions of melatonin on prolactin output exerted directly within the pituitary, thus providing an intrapituitary mechanism for the control of fertility (Fig. 10). The system operates in both long- and short-day breeders by fine-tuning the control of LH and FSH secretion and inducing complete suppression of the reproductive axis, respectively. The plasticity of the system is likely to rely on the input of the folliculostellate cell network and is crucial for the adaptation to the physiological requirements of the species.
